# Cooccurrences of Putative Endogenous Retrovirus-Associated Diseases

**DOI:** 10.1155/2017/7973165

**Published:** 2017-02-23

**Authors:** Christine Brütting, Alexander Emmer, Malte E. Kornhuber, Martin S. Staege

**Affiliations:** ^1^Department of Pediatrics I, Martin Luther University Halle-Wittenberg, Halle, Germany; ^2^Department of Neurology, Martin Luther University Halle-Wittenberg, Halle, Germany

## Abstract

At least 8% of the human genome is composed of endogenous retrovirus (ERV) sequences. ERVs play a role in placental morphogenesis and can sometimes protect the host against exogenous viruses. On the other hand, ERV reactivation has been found to be associated with different diseases, for example, multiple sclerosis (MS), schizophrenia, type 1 diabetes mellitus (T1D), or amyotrophic lateral sclerosis (ALS). Little is known about the cooccurrence of these diseases. If all these diseases are caused by ERV, antiretroviral therapy should perhaps also show some effects in the other diseases. Here, we summarize literature demonstrating that some ERV-associated diseases seem to appear together more often than expected, for example, MS and ALS, MS and T1D, MS and schizophrenia, or ALS and T1D. In contrast, some ERV-associated diseases seem to appear together less frequently than expected, for example, schizophrenia and T1D. Besides, some reports demonstrate amelioration of MS, ALS, or schizophrenia under antiretroviral therapy in human immunodeficiency virus-infected patients. If such results could be confirmed in larger studies, alternative therapy strategies for ERV-associated diseases like MS and schizophrenia might be possible.

## 1. Endogenous Retroviruses and Diseases

It is known that almost half of human DNA sequences belong to the group of repeated or (retro)transposable elements [1]. First discovered by McClintock in the 1950s [2], such sequences were considered as “junk” DNA for decades [3]. In the last years the perspective changed and retroelements are now seen as important drivers for species diversity [4] as well as possible factors in human diseases, especially in autoimmune diseases [5].

Endogenous retroviruses (ERV) have been detected in numerous eukaryotic organisms. Their exogenous counterparts have infected vertebrates including* Homo sapiens *[6]. At least 8% of the human genome is composed of endogenous retroviral sequences. These sequences were integrated into the human genome in the course of the evolution. The great majority of HERV are stabilized in the genome, but there is still ongoing or potential HERV genotype modification from parents to offspring throughout generations. As ERV are susceptible to mutations, the majority of ERV in our genome is not competent to replicate and most ERV sequences are presumably silent [7]. However, some of these sequences still have open reading frames (ORFs) and therefore have the potential to code for proteins or peptides [8, 9]. Oja and coworkers [10] found that about 7% of all ERV sequences in the human genome are transcriptionally active. ERV can be reactivated by some exogenous viruses like Epstein Barr virus (EBV), herpes simplex virus 1, or human immunodeficiency virus 1 [11–13]. Another possibility is the reactivation of ERV expression by hypoxia [14].

ERVs have also contributed to certain physiological genes through mutations and modifications that conferred physiologically important functions like placental morphogenesis [8] and can sometimes protect the host against infections with exogenous retroviruses [15]. On the other hand, ERV have also been found to be associated with different diseases [16, 17]. Our study addresses HERVs as being involved in the pathogenesis of chronic noninfectious diseases, for example, multiple sclerosis [18, 19], schizophrenia [20], type 1 diabetes mellitus [21], and amyotrophic lateral sclerosis [22] and, in parallel, as potential interfering factors when activated by infections with exogenous retroviruses such as HIV.

### 1.1. Multiple Sclerosis (MS)

Multiple sclerosis is a chronic immune-mediated inflammatory disease of the central nervous system with characteristic patchy demyelination. It is the most common chronic disabling CNS disease in young adults and affects about 2.3 million people around the world [23, 24]. The etiology of MS has not been completely decoded so far, supposing that causes are multifactorial including environmental influences [25] as well as epigenetic and genetic factors [26]. Family aggregation studies show that the general population prevalence of 0.1% [27] increases with the degree of kinship from 7–50-fold for biological first-degree relatives [28, 29] to a risk of about 300-fold for monozygotic twins [30, 31]. The impact of environmental factors on triggering of disease onset remains unclear. Sunlight exposure and resulting vitamin D levels [32] as well as EBV infection [33] are discussed.

Commonly an autoimmune attack against myelin autoantigens is considered as the main factor in the pathogenesis of MS [34, 35]. Additionally, HERVs are discussed to contribute to MS [36–38].

Several HERVs are considered to be involved in multiple sclerosis pathogenesis [39]. For example, the HERV-W* env* mRNA expression was selectively upregulated in brain tissue from individuals with multiple sclerosis as compared with controls [40].

### 1.2. Schizophrenia

Schizophrenia is a severe chronic psychiatric disorder characterized by “positive” symptoms like delusions and hallucinations as well as negative symptoms like blunted affect or emotional withdrawal [41]. Worldwide more than 23 million people suffer from schizophrenia [24]. The causes of schizophrenia are probably a mixture of genetic and environmental factors. The general population prevalence of about 1% increases with the degree of kinship from 6 to 13 % for biological first-degree relatives to a risk of 48% for monozygotic twins [42]. In addition to genetic factors, environmental factors like cannabis abuse [43] and urbanization [44] are discussed.

Schizophrenia seems to be associated with ERV. Elevated levels of multiple sclerosis-associated retrovirus (MSRV) and ERV-FRD sequences in the brains and cerebrospinal fluids of schizophrenia patients compared to healthy controls have been observed [45]. An increased transcription of HERV-W elements and the existence of antigens of HERV-W envelope and capsid proteins were found in blood samples from affected individuals [46]. In addition, a significantly higher HERV-K10 activity was detected in the brains of schizophrenia patients compared to healthy controls [47].

### 1.3. Type 1 Diabetes (T1D)

Type 1 diabetes is characterized by an autoimmune destruction of insulin producing beta cells in the pancreas which usually leads to absolute insulin deficiency [48]. It is one of the most common chronic diseases of childhood [49] although it can be diagnosed at any age. T1D has high and increasing worldwide prevalence rates with some regional variability [50]. Boys and men are more affected than girls and women [51]. The etiology of T1D is still unclear. Again, causes seem to be multifactorial including genetic factors like special HLA haplotypes [52] as well as environmental influences like vitamin D deficiency [53] and virus infections [54].

The association between T1D and endogenous retroviruses has been discussed controversially [55, 56]. There is some evidence for the association of HERV-K18 polymorphisms with T1D [57, 58]. Moreover, there seems to be a link between the retrovirus-like long-terminal repeat DQ-LTR13 and the genetic susceptibility to T1D [59].

### 1.4. Amyotrophic Lateral Sclerosis (ALS)

Amyotrophic lateral sclerosis is a neurodegenerative disease characterized by the loss of spinal and cortical motor neurons [60]. It is an often rapidly progressive disease with a median survival of 3 years [61]. ALS has a prevalence of around 6 per 100,000 [62]. Men are a little bit more often affected than women with a male-female ratio of 1.5 to 1 [63]. Most cases of ALS (90–95%) are sporadic, only about 5–10% of ALS cases are familial [64]. In familial ALS various genes have been identified that are associated with the disease. Superoxide dismutase 1 (*SOD1*) is the most important of them, accounting for up to 20% of all familial ALS cases [65, 66].

For ALS there are some associations to ERV. HERV-K transcripts are increased in autopsy brain tissue of patients with ALS compared to controls [22] and antiretroviral seroreactivity in patients with sporadic ALS was found [67]. Thereby neuronal TAR DNA binding protein 43 (TDP-43) regulates endogenous retrovirus-K viral protein accumulation [68]. Expression of HERV-K or its envelope protein in human neurons caused retraction and beading of neurites [69]. This is also reproducible in transgenic mouse model: expressing the envelope gene of HERV-K leads to progressive motor neuron dysfunction accompanied by selective loss of volume of the motor cortex, decreased synaptic activity in pyramidal neurons, dendritic spine abnormalities, nucleolar dysfunction, and DNA damage [69].

### 1.5. Human Immunodeficiency Virus (HIV) Infection

HIV infection and acquired immune deficiency syndrome (AIDS) comprise a wide range of disorders and diseases caused by HIV, including tumors or opportunistic infections [70, 71]. Likely more than 35 million people around the world have been diagnosed with HIV [72]. Women are a little bit more often affected than men [73].

The HIV infection is a special case in our register of diseases that seem to be associated with ERV as HIV itself is a retrovirus. HIV can be divided into two virus subtypes (HIV-1 and HIV-2) which infect and kill CD4+ T lymphocytes [74]. The treatment consists of highly active antiretroviral therapy (HAART) which slows the progression of the disease and normally leads to a much higher life expectancy than without the therapy [75]. Interestingly, HIV infection seems to be associated with ERV reactivation. Vincendeau and coworkers [76] detected in three persistently HIV-1 infected cell lines an increased transcription of HERV-E, HERV-T, HERV-K, and ERV9. HERV-K102 is often activated in cases of HIV viremia in contrast to healthy controls [77]. Expression of human endogenous retrovirus type K (HML-2) is activated by the Tat protein of HIV-1 [78, 79].

## 2. Cooccurrence of ERV-Associated Diseases

We searched the literature for cooccurrences of the diseases discussed above.  Table 1 summarizes publications [80–157] that investigated such cooccurrences.

### 2.1. MS and Schizophrenia

The cooccurrence of MS and schizophrenia has considered being a very rare event taking into account the few case reports in the literature [81, 82, 86]. Nevertheless, Jongen [83] suggested MS screening in patients with psychotic disorder even if only slight neurological abnormalities are present. The first epidemiologic evidence for a positive association between MS and schizophrenia was shown in a Canadian population including 2.45 million people [84]. This association was also found in Taiwanese [87] and Danish [88] patients with MS. In a nationwide Swedish cohort [90] and a Canadian population [89] there was an association between MS and bipolar disorders as well as between MS and depression; in contrast there was no or even a negative association between MS and schizophrenia. Also a Danish cohort study failed to find a positive association [85]. Another Danish cohort study found at least a higher risk for parents and siblings of schizophrenia patients for getting MS [158]. Brodziak and coworkers [159] formulate a theory that ERV causing MS and schizophrenia are activated during pregnancy by some infection of the mother.

### 2.2. MS and T1D

Several studies have analyzed the cooccurrence of MS and type 1 diabetes [91–96]. Most studies found higher incidence rates [96] or prevalence rates [91, 94] between both diseases than expected. Particularly adult women with type 1 diabetes have a dramatically higher risk of getting MS [95].

Both diseases have some features in common like the similar geographical distribution and increased family risk [160, 161]. Besides, susceptibility to both disorders is associated with common variants of the HLA-*DRB1* and -*DQB1* loci [162]. In contrast, an inverse risk-association between MS or T1D and distinct HLA alleles is discussed: HLA alleles known to confer to T1DM (DRB1*∗*0401, DRB1*∗*0404, DQB1*∗*0302, DRB1*∗*0301, and DQB1*∗*0201) rarely occur in MS patients. HLA susceptibility genes for T1DM (DRB1*∗*1501, DQB1*∗*0602-DQA1*∗*0102) predispose to MS [163]. Accordingly, our results are of special interest.

### 2.3. MS and ALS

The concurrence of MS and ALS was supposed to be extremely rare [100, 104, 105]. One reason might be that for a long time most detected cases were published as case reports [97–99, 101, 102]. In addition, large datasets were scanned for the simultaneous occurrence of MS and ALS [103, 108]. Taking all data together, it seems that the concurrence of MS and ALS is higher than expected [106, 107]. This association was also found for first-degree kinship [164, 165].

### 2.4. MS and HIV Infection

Considering the rare number of case reports [110, 114], the cooccurrence of MS and HIV infection seems to be an uncommon event. A first population-based register study [111] including 5,018 HIV patients and 50,149 controls failed to find an association (possibly due to the relatively small number of both groups) but showed a negative trend between both diseases. When increasing the number of both groups to 21,207 HIV positive patients and 5,298,496 controls significant negative association between both diseases was observed [113].

Some of the case reports analyzed patients who were treated with antiretroviral therapy. In the consequence, all of them showed less MS related deficits for over years [109, 112, 115]. Therefore, it seems imaginable that the treatment of HIV infection is coincidentally also treating or preventing the progression of MS [111, 113].

### 2.5. Schizophrenia and T1D

There are only few studies considering the cooccurrence of schizophrenia and type 1 diabetes (Table 1) on which 2 studies failed to find an association [85, 117] and 2 studies found a negative association between both diseases [116, 118].

In contrast, there is a higher prevalence of T1D in first-degree relatives of schizophrenia patients [85, 166, 167].

### 2.6. Schizophrenia and ALS

There are several case reports about the simultaneous occurrence of schizophrenia and ALS [119–126]. Most of them are historically old and the diagnosis is sometimes potentially questionable [168]. We failed to find any register-based study about patients with both diseases. At least there is one study showing the elevated risk for ALS in relatives of schizophrenic patients [169]. As patients with schizophrenia have a nearly threefold increase in overall mortality compared to controls [170], they might have a reduced chance of getting ALS because ALS usually occurs after the age of 50 [168].

### 2.7. Schizophrenia and HIV Infection

The cooccurrence of schizophrenia and HIV infection is described very often [127–132, 134, 136–138]. Susser and coworkers [133] reported a patient where HIV infection was determined before the onset of schizophrenia. Reasons for this coincidence were assumed to be mainly sexual activity accompanying high risk behavior [171, 172] and substance misuse with contaminated equipment and shared needles [173, 174]. Stewart and coworkers [131] observed that in Maryland, USA, 5.9% of patients with schizophrenia were HIV positive tested whereas the prevalence of HIV in the overall population in Maryland was about 0.032% [175]. Interestingly, there is also a higher risk for patients with HIV of getting schizophrenia and this elevated risk decreased after antiretroviral therapy [139].

### 2.8. Type 1 Diabetes and ALS

Unfortunately, several studies about diabetes and ALS do not distinguish between type 1 and type 2 diabetes [176, 177]. One problem is that the authors often do not have the possibility of distinguishing between both types [178] as some misclassification of hospital data is not avoidable. The very few studies of classified type 1 diabetes and ALS have shown inconsistent results [179]. Mariosa and coworkers [140] failed to detect more cases of type 1 diabetes in ALS patients compared to healthy controls whereas Turner and coworkers [107] showed an accumulation of insulin-dependent diabetes in ALS patients. Taking into account the age at ALS and diabetes diagnosis, there is an association between ALS and diabetes when patients are younger indicating that these diabetes cases are type 1 diabetes cases [140, 178]. All in all some authors speculate that type 1 diabetes increases the risk of getting ALS, while type 2 diabetes shows some protective effects [180].

### 2.9. Type 1 Diabetes and HIV Infection

There are lots of case reports about patients with type 1 diabetes as well as HIV infection [141–148, 150, 152]. This is not surprising as both diseases appear quite often in the world [50, 72].

In spite of the relatively frequent occurrence of both diseases there are no analyses about the kind of association between them. The only two register-based studies [149, 151] did not check for that.

### 2.10. ALS and HIV Infection

Unfortunately the classification between clinically definite ALS and ALS-like syndromes or disorders is often not very clear in the literature about patients with HIV so that most cases were clinically probable or possible cases of ALS [157]. Only 6 clinically definite ALS cases in patients with HIV infection have been found [153–157].

Interestingly, ALS [154, 155] and ALS-like disorders [181] in patients with HIV infection seem to respond very well to antiretroviral therapy and symptoms can disappear completely.

## 3. Conclusions

Some ERV-associated diseases seem to appear together more often than expected: MS and ALS, MS and T1D, MS and schizophrenia, schizophrenia and HIV infection, or ALS and T1D. On the other hand, some ERV-associated diseases seem to appear together less frequently than expected: MS and HIV infection or schizophrenia and T1D. Besides, amelioration of MS, ALS, and schizophrenia under antiretroviral therapy in HIV infected patients has been observed. Up to now there are mainly case reports for such effects available. One study compared HERV-K titers in HIV infected patients receiving successful highly active antiretroviral therapy (HAART) versus unsuccessful HAART. In this study, titers were undetectable in the first group and persistently higher in the other group [182]. All in all there is insufficient data about the cooccurrence of ERV-associated diseases and their response to antiretroviral therapy. In our opinion it would be reasonable to check HIV patients with cooccurring diseases regarding amelioration of these diseases under antiretroviral therapy in large register studies. If the positive results could be confirmed, then alternative therapy for putative ERV-associated diseases seems to be possible.

## Figures and Tables

**Table 1:** Cooccurrence of diseases with possible involvement of ERV reactivation.

Number of cases	Association between diseases	Comments	Ref.
*MS and schizophrenia*			
1	n.a.^(1)^	Case report	[80]
2	n.a.^(1)^	Case report	[81]
10	n.a.^(1)^	Research study	[82]
1	n.a.^(1)^	Case report	[83]
n.a.^(2)^	Positive	Epidemiological investigation	[84]
3	No association	Population-based register study	[85]
1	n.a.^(1)^	Case report	[86]
67	Positive	Population-based controlled study	[87]
63	Positive	Population-based register study	[88]
39^(3)^	No association	Epidemiologic investigation	[89]
36	Negative	Population-based register study	[90]

*MS and T1D*			
5	Positive	Population-based register study	[91]
3	n.a.^(1)^	Clinical register study	[92]
1	No association	Clinical register study	[93]
28	Positive	Cohort study	[94]
n.a.^(4)^	Positive	Clinical register study	[95]
11	Positive	Population-based cohort study	[96]

*MS and ALS*			
1	n.a.^(1)^	Case report	[97]
1	n.a.^(1)^	Case report	[98]
1	n.a.^(1)^	Case report	[99]
1	n.a.^(1)^	Case report	[100]
1	n.a.^(1)^	Case report	[101]
1	n.a.^(1)^	Case report	[102]
1	n.a.^(1)^	Research study	[103]
1	n.a.^(1)^	Case report	[104]
1	n.a.^(1)^	Case report	[105]
7	Positive	Epidemiologic investigation	[106]
143	Positive	Clinical register study	[107]
1	No association	Population-based case-control study	[108]

*MS and HIV infection*			
1	n.a.^(1)^	Case report	[109]
1	n.a.^(1)^	Case report	[110]
1	No association	Population-based register study	[111]
1	n.a.^(1)^	Case report	[112]
10	Negative	Comparative cohort study	[113]
1	n.a.^(1)^	Case report	[114]
1	n.a.^(1)^	Case report	[115]

*Schizophrenia and T1D*			
0^(5)^	Negative	Population-based register study	[116]
n.a.^(6)^	No association	Population-based register study	[117]
24	No association	Population-based register study	[85]
24	Negative	Population-based register study	[118]

*Schizophrenia and ALS*			
2	n.a.^(1)^	Case report	[119]
1	n.a.^(1)^	Case report	[120]
2	n.a.^(1)^	Case report	[121]
1	n.a.^(1)^	Case report	[122]
1	n.a.^(1)^	Case report	[123]
2	n.a.^(1)^	Case report	[124]
2	n.a.^(1)^	Case report	[125]
2	n.a.^(1)^	Case report	[126]

*Schizophrenia and HIV infection*			
13	n.a.^(1)^	Clinical register study	[127]
3	n.a.^(1)^	Clinical cohort	[128]
8	n.a.^(1)^	Cohort study	[129]
11	n.a.^(1)^	Clinical cohort	[130]
10	n.a.^(1)^	Clinical cohort	[131]
13	n.a.^(1)^	Clinical cohort	[132]
1	n.a.^(1)^	Clinical cohort	[133]
476	n.a.^(1)^	Population-based register study	[134]
7	n.a.^(1)^	Clinical cohort	[135]
11	n.a.^(1)^	Clinical cohort	[136]
1	n.a.^(1)^	Case report	[137]
28	n.a.^(1)^	No data	[138]
68^(7)^	Positive	Population-based cohort study	[139]

*T1D and ALS*			
216	Positive	Clinical register study	[107]
43	No association	Population-based case-control study	[140]

*T1D and HIV infection*			
1	n.a.^(1)^	Case report	[141]
1	n.a.^(1)^	Case report	[142]
1	n.a.^(1)^	Case report	[143]
1	n.a.^(1)^	Case report	[144]
1	n.a.^(1)^	Case report	[145]
1	n.a.^(1)^	Case report	[146]
1	n.a.^(1)^	Case report	[147]
1	n.a.^(1)^	Case report	[148]
7	n.a.^(1)^	Clinical register study	[149]
3	n.a.^(1)^	Case report	[150]
10	n.a.^(1)^	Population-based register study	[151]
1	n.a.^(1)^	Case report	[152]

*ALS and HIV infection*			
1	n.a.^(1)^	Case report	[153]
1	n.a.^(1)^	Case report	[154]
1	n.a.^(1)^	Retrospective hospital cohorts	[155]
1	n.a.^(1)^	Case report	[156]
2	n.a.^(1)^	Case report	[157]

^(1)^Not available, not tested, or not presented in the publication.

^(2)^The number of cases was not reported; the prevalence of psychotic disorders among more than 10,000 MS patients was reported as 1.3%.

^(3)^The number of cases was inferred from incidence as presented in the publication; the number was not explicitly reported.

^(4)^Not available; the number of patients was not reported.

^(5)^No schizophrenia cases were found among 1,154 diabetics below 27 years of age.

^(6)^Not available; the number of cases was not reported; the prevalence of psychoses among nearly 17,000 patients with T1D was reported as 0.8%.

^(7)^Number of HIV positive patients developing schizophrenia.
